# Distinct Roles of Bcl-2 and Bcl-Xl in the Apoptosis of Human Bone Marrow Mesenchymal Stem Cells during Differentiation

**DOI:** 10.1371/journal.pone.0019820

**Published:** 2011-05-12

**Authors:** Lisa Oliver, Erika Hue, Julien Rossignol, Gwenola Bougras, Philippe Hulin, Philippe Naveilhan, Dominique Heymann, Laurent Lescaudron, François M. Vallette

**Affiliations:** 1 INSERM, UMR 892, Équipe Labellisée «Ligue contre le Cancer», Nantes, France; 2 Faculté de Médecine, Université de Nantes, Nantes, France; 3 INSERM, UMR 643, Nantes, France; 4 Cellular and Tissular Imaging Core Facility (MicroPICell), Université de Nantes, Nantes, France; 5 INSERM, UMR 957, Nantes, France; 6 Service de Physiologie Animale et Humaine, UFR Sciences et Techniques, Université de Nantes, Nantes, France; Albert Einstein Institute for Research and Education, Brazil

## Abstract

**Background:**

Adult mesenchymal stem cells (MSCs) can be maintained over extended periods of time before activation and differentiation. Little is known about the programs that sustain the survival of these cells.

**Principal Findings:**

Undifferentiated adult human MSCs (hMSCs) did not undergo apoptosis in response to different cell death inducers. Conversely, the same inducers can readily induce apoptosis when hMSCs are engaged in the early stages of differentiation. The survival of undifferentiated cells is linked to the expression of Bcl-Xl and Bcl-2 in completely opposite ways. Bcl-Xl is expressed at similar levels in undifferentiated and differentiated hMSCs while Bcl-2 is expressed only in differentiated cells. In undifferentiated hMSCs, the down-regulation of Bcl-Xl is associated with an increased sensitivity to apoptosis while the ectopic expression of Bcl-2 induced apoptosis. This apoptosis is linked to the presence of cytoplasmic Nur 77 in undifferentiated hMSCs.

**Significance:**

In hMSCs, the expression of Bcl-2 depends on cellular differentiation and can be either pro- or anti-apoptotic. Bcl-Xl, on the other hand, exhibits an anti-apoptotic activity under all conditions.

## Introduction

Self-renewal and limited proliferation are the processes that allow the maintenance of the stem cell pools throughout life [1]. Stem cells must thus maintain their molecular blueprints over long periods of time and this quality control is achieved, in part, through the induction of cell death in damaged cells [2]. Mesenchymal stem cells or bone marrow stromal cells (MSCs) are committed to mesenchymal cell lineages such as bone, cartilage, tendon, ligament, adipocytes and muscle; and possibly to other cell types such as neurons [3]–[5]. MSCs are also essential for the proliferation and differentiation of hematopoietic cells within the bone marrow compartment [3]–[5]. Beyond their transdifferentiation process, MSCs are also involved in tissue repair and recently have been considered as an ideal therapeutic vehicle in many diseases [6]–[7].

One important feature of MSCs is their capacity to survive over long periods of time under homologous conditions but to die rapidly upon their transfer into another individual [3]–[5]. These observations suggest that these cells, which are highly proliferative *in vitro*, also possess an efficient cell death machinery. There is little information about the mechanism of survival of most adult stem/progenitor cells, although this question must be crucial with regards to their physiological role(s). Apoptosis is a cell death program that is instrumental in fetal and adult tissue homeostasis. It has been shown that fetal MSCs exhibit functional apoptotic pathways [8]. In agreement with the latter hypothesis, genetically modified rat MSCs containing an anti-apoptotic Bcl-2 gene exhibited enhanced cell survival upon intracardiac engraftment [9]. On the other hand, it has been shown that MSCs can survive pro-apoptotic anti-cancer treatments [10]–[11], suggesting that MSCs are highly resistant to radio-or chemo-induced cell death.

In this study, we have addressed the question of the control of cell death in these cells. We found that undifferentiated hMSCs were highly resistant to apoptosis while differentiation, even at the very early stages, was accompanied by an increase in their sensitivity to apoptosis. This process is differentially controlled by members of the BCL-2 family of proteins, which are instrumental in the induction of apoptosis [12] but have been shown to display distinct roles during hematopoiesis [13].

## Materials and Methods

### Materials

Unless stated otherwise, all cell culture material was obtained from Gibco (Invitrogen, Cergy Pontoise, France) and all chemicals were from Sigma-Aldrich (St. Louis, MO, USA). The 14 bone marrow samples used in this study were obtained from healthy donors operated at the Dept of Orthopedics at “Centre Hospitalier Universitaire de Nantes”. The average age of patients was 41±3 yrs (ranging from 25 to 56 yrs, 6 males and 8 females). Human fibroblast cultures were obtained from foreskins obtained from the Dept of Pediatric at “Hôpital Mère et Enfant de Nantes”.

### Methods

#### Ethics statements

Patient data were obtained and handled according to French laws and recommendations of the French National Commitee of Ethics (Comité Consultatif National d'Ethique pour les Sciences de la Vie et de la Santé).

#### Cell Culture

The bone marrow cells were isolated by density gradient centrifugation (Ficoll). The cells collected at the interface were cultured in alpha-MEM modified with ribonucleosides and deoxyribonucleosides supplemented with 20% fetal calf serum, with 2 mM L-glutamine, 100 U/mL penicillin, 100 µg/mL streptomycin (complete medium) in an atmosphere of 5% CO_2_ and 95% humidity at 37°C. MSC cultures were used between passages 2 and 10. Cultures were kept at about 75% confluence and passaged every 5–7 days. Mincing the tissue and trypsin treatment isolated human foreskin fibroblasts. The fibroblasts recuperated were cultured in RPMI-1640 supplemented with 10% fetal calf serum, 2 mM L-glutamine, 100 U/mL penicillin, 100 µg/mL streptomycin in an atmosphere of 5% CO_2_ and 95% humidity and used between passages 2 and 35. The K562 cells were cultured in RPMI supplemented with 10% fetal calf serum, 2 mM L-glutamine, 100 U/mL penicillin, 100 µg/mL streptomycin and Glioma primary cultures (GBM) were cultured in DMEM supplemented with 10% fetal calf serum, 2 mM L-glutamine, 100 U/mL penicillin, 100 µg/mL streptomycin, both in an atmosphere of 5% CO_2_ and 95% humidity at 37°C. Cell viability was determined by Trypan blue exclusion on a minimum of 200 cells using a Countess automated cell counter (Invitrogen).

#### Hypoxic treatment

Cells were cultured in complete medium in an atmosphere of 3% O_2_, 5% CO_2_ and 95% humidity at 37°C in a hypoxia chamber (Invivo 400, 3M, France).

#### Differentiation of MSC

Osteogenic MSC differentiation was induced *in vitro* by culturing in NH OsteoDiff medium (Miltenyi Biotec GmbH, Bergisch Gladbach, Germany) over 21 days. Osteogenic differentiation was detected by the expression of alkaline phosphatase using the 5-Bromo-4-chloro-3-indolyl phosphate/Nitro blue tetrazolium (BCIP/NBT) substrate (Sigma*fast* B5655) according to the manufacturer's instructions. Adipocytes differentiation was induced *in vitro* by culturing the cells in NH AdipoDiff Medium (Miltenyi Biotec France) over 21 days. Adipocyte differentiation was detected by coloration with Oil Red O, which colors hydrophobic lipids. Neural transdifferentiation was induced in hMSCs by culturing the cells for 48 h in complete medium containing 20 ng/ml human recombinant (hr) bFGF (100-18B, PeproTech, France) and 20 ng/ml hrEGF (100-15, PeproTech, France). The cells were then cultured in complete medium containing 10 ng/ml hrBDNF (Sigma, B-3795) to induce differentiation along the neuronal pathway.

#### Transfection and viral infection

hMSCs (10^6^) were nucleofected with 2 µg plasmid: pGFPmax, pRcCMV (pCMV) empty or containing the Bcl-2 insert (pBcl-2) using the Amaxa human MSC nucleofector kit (Lonza, Levallois-Perret, France). After 16 h post-transfection the cells were used in experiments. MSC were cultured with lentiviral particles (Sigma-Aldrich) at a multiplicity of infection of 15 in complete medium for 48 h. Lentiviral particles used for the knock-down of Bcl-Xl were: TRCN0000033499, TRCN0000033500, **TRCN0000033501**, TRCN0000033502, TRCN0000033503 and for Nur 77: **TRCN0000019425**, TRCN0000019426, TRCN0000019427, TRCN0000019428. The initial experiments were done with the set of viral particles; however, the repeat experiments were done with those in bold.

#### FACScan Analysis

The phenotype of MSC was monitored by flow cytometry. For phenotypic analysis, conjugated antibodies were used (cf. **Table S1**). Briefly, 2×10^5^ cells were resuspended in complete medium for 30 min at 4°C. For intracellular staining, cells were fixed with 4% paraformaldehyde for 10 min and permeabilized in PBS containing 0,5 % saponin. The cells were incubated with the primary antibody for 30 min at 4°C in PBS, 0,25% saponin and then, where necessary, the secondary antibody was added for 30 min at 4°C. Cells were washed twice in PBS before analyzed on a FACScalibur (BD Biosciences, Le Pont de Claix, France) using Cell Quest Pro software. The appropriate isotype controls were included and a minimum of 10 000 events were acquired for each condition. The debris was excluded from the analysis according to their FSC/SSC proprieties. BD ApoAlert APO 2.7-PE (BD Biosciences) was used to determine the percentage of apoptotic cells according to the manufacturer's instructions.

#### Quantification of caspase activation

Cells were lysed vol:vol in Nonidet P-40 (NP-40) lysis buffer (142.5 mM KCl, 5 mM MgCl_2_, 10 mM HEPES [pH 7.2], 1 mM EDTA, 0.25% NP-40, 0.2 mM PMSF, 0.1% aprotinin, 1 µg/ml pepstatin and 1 µg/ml leupeptin) at 4°C for 30 min. The protein concentrations were quantified and the caspase activity was quantified using the fluorogenic substrate Ac-DEVD-AMC as described by the manufacturer.

#### Time-lapse analysis

Time-lapse video-microscopy experiments were performed using a Zeiss Axiovert 200-M inverted microscope (Carl Zeiss, Le Pecq, France) and the AxioVision 4.6 program. Dishes were placed inside an Incubator XL-3, on a heating insert M06 (37°C) topped with a CO_2_-cover HM connected to a CO_2_ controller that maintained the environmental CO_2_ concentration at 5% for the duration of filming. Digital pictures were acquired and saved every 10 min over 48 h using an AxioCam MR digital camera. The series of photographs were displayed as continuous time-lapse movies for analyses. Cell death was quantified at each acquisition and represented graphically as number of cell death every hour over the 48 h.

#### Western blots

Total proteins were extracted in 1% NP-40, 0,5% sodium-deoxycholate, 0,1% SDS in PBS supplemented with protease inhibitor cocktail from Roche Diagnostics (Mannheim, Germany). Protein concentration was determined using Bradford assay (Biorad, Hercules, CA, USA). Protein extracts were separated on SDS-PAGE, transferred onto PVDF membrane (Millipore, St. Quentin-Yvelines, France) and revealed with ECL (Roche Diagnostics). Primary antibodies were used at 1/1000 dilution: mouse monoclonal anti-actin (MAB1501R, Millipore), rabbit polyclonal anti-Bax (epitope: aa43–61, DakoCytomation, Trappes, France), mouse monoclonal anti-Bax^2D2^ (epitope: aa3-16, Beckman-Coulter, Fullerton, CA, USA), mouse monoclonal anti-Bax^6A7^ (epitope: aa12-24, Beckman-Coulter), rabbit monoclonal anti-Bcl-x (1018-1, Epitomics, France), rabbit monoclonal anti-Bcl-2 (1017-1, Epitomics, France), rabbit monoclonal anti-caspase 3 (sc-7272, Santa Cruz Biotech, Ca, USA), rabbit polyclonal anti-Nur 77 (sc-5569, Santa Cruz), HRP-conjugated secondary antibodies were from Biorad. Quantification was performed with the software ImageJ.

#### Immunocytochemistry

Cells were grown on gelatin-coated glass cover-slips. Cells were fixed in 4% paraformaldehyde for 20 min, permeabilized with 0,1% SDS for 10 min, blocked with 3% BSA for 20 min, and then incubated with primary antibodies for 1 h followed by secondary Alexa antibodies (Molecular Probes-Invitrogen) for 1 h. Cells were finally mounted with mowiol polymerizing solution. Primary antibodies were: rabbit polyclonal anti-caspase 3_active_ (2305-PC-100, Trevigen, Interchim, France), mouse monoclonal anti-F1-ATPase (Molecular Probes, Invitrogen) and rabbit polyclonal anti-F1-ATPase a gift from Pr J. Lunardi. The cells were analyzed under an inverted Leica TCS-SP1 confocal microscope. A 63×1.4 NA oil objective and sequential scanning was used. The 3D reconstitution was done using the Amira 3D v4.1.1 Imaging program. The quantification of the co-localization was done using the Metamorph v7.5.6. Program.

#### Proximity ligation assay (PLA)

This assay enables the detection and quantification of protein interactions using secondary antibodies labeled with a pair of oligonucleotides that will generate a signal when the two PLA probes are in close proximity (Olink Bioscience, Uppsala, Sweden). The signal is detected as an individual fluorescent dot. Briefly, the cells were cultured overnight on gelatinized glass coverslips. Cells were fixed in 4% paraformaldehyde for 15 min, permeabilized with 0,25% Triton X-100/PBS for 5 min and blocked in 1× blocking solution for 30 min. Cells were incubated with primary antibodies polyclonal rabbit anti-Nur 77 and monoclonal mouse anti-Bcl-2 diluted in blocking solution overnight at 4°C and then with secondary antibodies conjugated with oligonucleotides (PLA anti-mouse MINUS and PLA anti-rabbit PLUS) diluted in blocking solution for 2 h at 37°C. This was followed by hybridization, ligation, amplification and detection. The distance between the two primary antibodies must be less than 40 nm to generate a signal, which results in a highly specific assay to detect for protein-protein complexes. The slides were mounted using Prolong® Gold anti-fade reagent with Dapi (Invitrogen) and then analyzed under the confocal microscope. A 63×1.4 NA oil objective and sequential scanning with filters 420–480 nm for Dapi and 560–615 nm for fluorophore Tye-563 were used.

## Results

### Characterization of hMSCs

**Figure 1:**
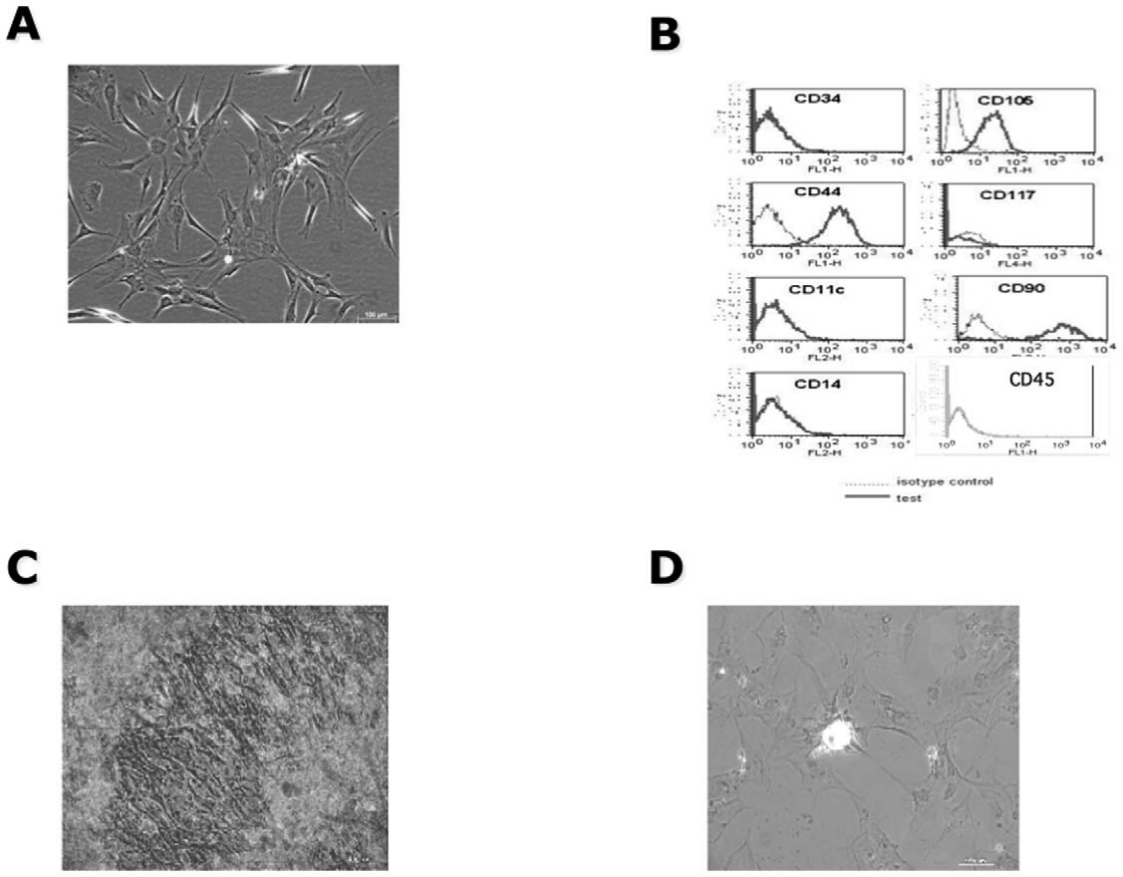
Characterization of *in vitro* hMSCs. (**A**) Cell morphology: Photograph of hMSCs cultured in complete medium as described in materials & methods. Note the fibroblast-like morphology of the cells. (**B**) Markers (cytometry): Results are represented as FACScan histograms; the gray line corresponds to the isotype control and the solid black line to the specific antibody tested. Data shown are representative of at least three independent experiments. (**C, D**) hMSCs were cultured for 3 weeks in either osteogenic (**C**) or adipogenic (**D**) differentiation media. Matrix mineralization was determined by alkaline phosphatase staining (**C**) and lipid vacuoles were stained with Oil Red O (**D**) as described in experimental procedures. The cultures were visualized under an upright Nikon TMS microscope, magnification ×20.

MSCs were isolated from human bone marrow samples as described by Pittenger *et al.* [14]. After a few days, the cultures became enriched with fibroblast-like cells that became predominant after 2 weeks of culture (**Figure 1A**). The cell population was characterized by flow cytometric analysis for the expression of CD105, CD90 and CD44 markers (**Figure 1B**). There was no detectable contamination of hematopoietic cells since the cells were negative for markers of the hematopoietic lineage, including the lipo-polysaccharide receptor CD14, CD34, and the leukocyte common antigen CD45 (**Figure 1B**). The cells were able to differentiate into several lineages *in vitro*, including osteoblasts and adipocytes (**Figures 1C and D**). Our findings are in agreement with previous reports and support the conclusion that our population is enriched with hMSCs [15]–[16]. Human MSCs cease to grow after about 20–30 population doublings due to senescence [17]–[18]. The most common characteristics of a senescent phenotype are a gradual decrease in the proliferation potential and a decrease in differentiation capacity [19]–[20]. Thus, to avoid the presence of senescent cells but also that of contaminant or differentiated cells, we determined that hMSCs should be plated at an initial concentration of 5×10^4^ to 10^5^ and used only between passages 2 and 10.

### Undifferentiated hMSCs are resistant to apoptosis

**Figure 2:**
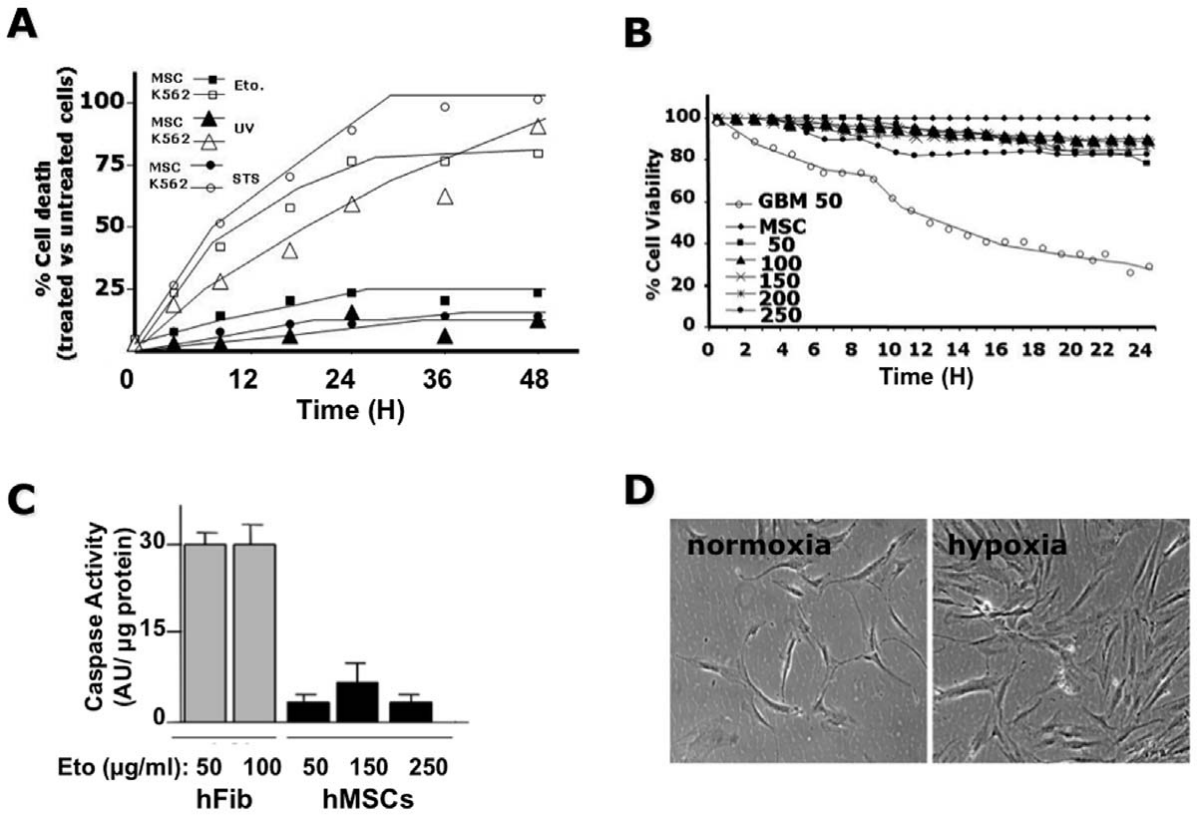
Absence of cell death in hMSCs. (**A**) Induction of cell death using staurosporine (STS), UV-irradiation and etoposide (Eto) in K562 cells and hMSCs. The cells were plated at 5×10^4^ cells/ml in 24-well plates. 24 h later the different apoptosis inducing agents were added to the culture medium and the cells were analyzed over 48 h using video-microscopy with an acquisition every 10 min. The number of dead cells was determined at each time point and rounded up for every hour. The results are presented as the percentage of dead cells in treated cultures versus untreated cultures. The number of cells analyzed was about 100 per condition. The results are representative of three independent experiments and of 3 different hMSC cultures. (**B**) Effect of soluble Fas ligand (sFasL) on hMSCs or GBM primary cultures. Quantification of the viability of hMSCs cultured in the absence or in the presence of increasing concentrations (50, 100, 150, 200 and 250 ng/ml) sFasL and GBM cells cultured in the presence of 50 ng/ml sFasL was determined by time-lapse microscopy over 24 h using 10 min intervals. Cell viability was determined at every acquisition as described in (**A**. **C**) Human MSCs or fibroblasts (hfib) were cultured in the absence or in the presence of etoposide (Eto) at the indicated concentrations over 24 h. The cells were then lysed and the caspase activity determined in 20 µg cell extract using Ac-DEVD-AMC as a substrate. The results are expressed as arbitrary units of caspase activity per µg protein. (**D**) Effect of hypoxia on hMSCs survival. Human MSCs were cultured for 48 h under normoxia (20% O_2_) or hypoxia using a hypoxia work-station at 3% O_2_, 5% CO_2_ in 95% humidified air. Note that experiments shown are representative of hMSCs obtained from at least five different donors.

Contrary to the huge body of data available on the control of life and death of hematopoietic stem cells, very little data are available on the mechanisms of survival used by hMSCs [21]. It has been shown that MSCs were resistant to chemotherapy-induced apoptosis [10]–[11], but the actual mechanism(s) of resistance of these cells to the cell death program were not determined. Previous data have shown that fetal MSCs are sensitive to both intrinsic mitochondrial and extrinsic receptor-mediated apoptotic pathways [8]. We thus examined the response of the isolated hMSCs to various cell death inducers such as staurosporine (STS), a broad kinase inhibitor, UV-irradiation and etoposide (Eto), the latter acting mostly through DNA damage-induced apoptosis [22]. As shown in **Figure 2A**, none of these treatments triggered cell death in hMSCs while K562, an erythroleukemia cell line was sensitive to these cell death inducers. It should be added that a dose response effect of etoposide and staurosporine was done and the effect of the optimal dose was used to determine the response to cell death over time (**data not shown**). We conclude from these results that hMSCs are resistant to cell death mediated mainly through the mitochondrial apoptotic pathway.

Next, we investigated the response of these cells to the extrinsic receptor-mediated apoptotic pathway triggered by soluble Fas ligand (sFasL). Time-lapse experiments over 24 h showed less than 10% cell death to concentrations of up to 200 ng/ml sFasL (**Figure 2B**). Thus, even at very high concentrations of sFasL, hMSCs did not undergo apoptosis while at a much lower concentration (i.e. 50 ng/ml) sFasL induced apoptosis in human Glioma (GBM) primary cultures (**Figure 2B**), as previously described [23]. Next, using caspase activity, a landmark of apoptosis, we compare the sensitivity toward etoposide-induced apoptosis in hMSCs to that of primary cultures of human foreskin fibroblasts (see experimental procedures). As shown in **Figure 2C**, hMSCs were resistant to caspase activation by etoposide while human fibroblasts exhibited maximum caspase activation at the lowest concentration. As hypoxia and serum-deprivation induced apoptosis in rat MSCs [24], we examined the effect of hypoxia on the survival of hMSCs. As shown in **Figure 2D**, hypoxia did not induce apoptosis in these cells but on the contrary, it triggered proliferation as previously described [25].

### Lack of cytochrome c release accounts for resistance to apoptosis in undifferentiated hMSCs

**Figure 3:**
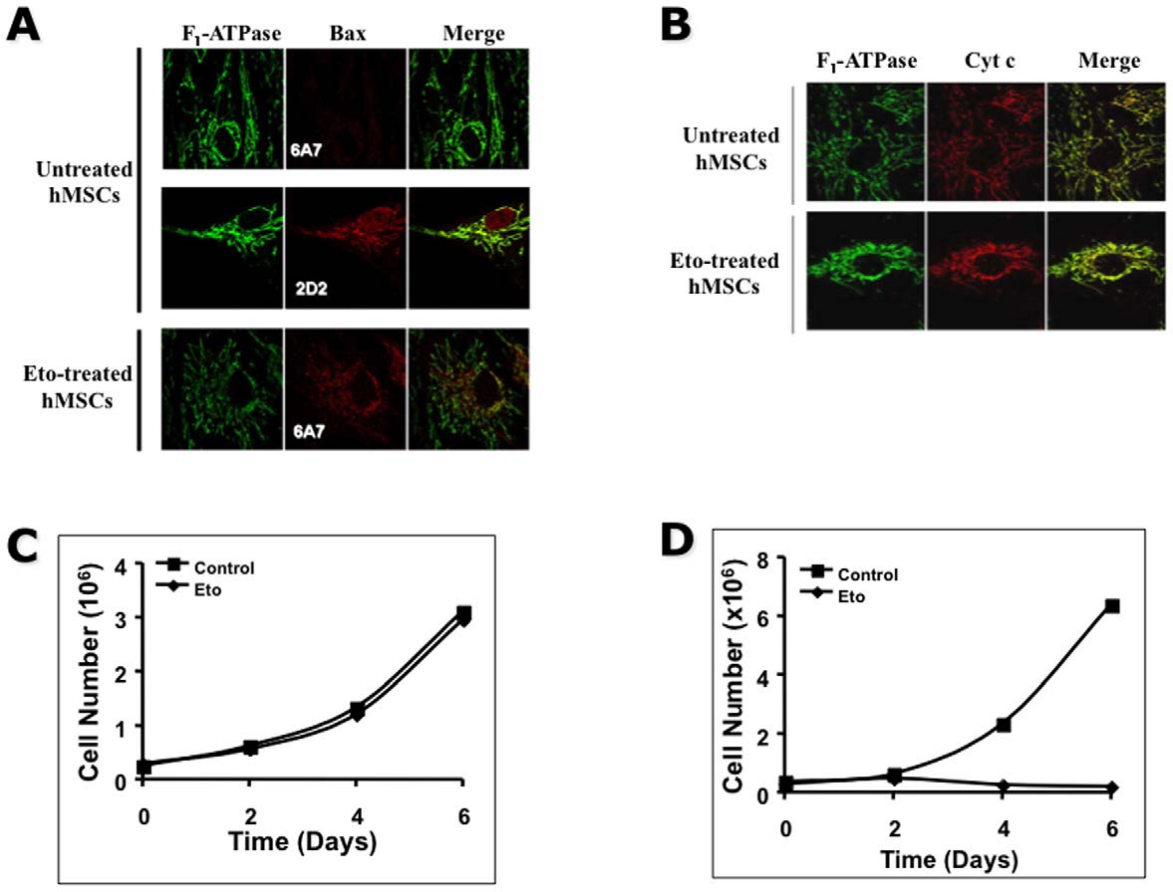
Absence of release of cyt c from mitochondria despite the activation of Bax in hMSCs. (**A, B**) Laser confocal analyses of hMSCs after etoposide treatment. Cells were cultured in the absence (untreated) or in the presence of 50 µg/ml Etoposide (Eto) for 24 h then labeled with polyclonal anti-F_1_-ATPase (mitochondria: green) and either (**A**) monoclonal anti-Bax^2D2^ (2D2: recognizes all forms of Bax: red) and/or anti-Bax^6A7^ (6A7: recognizes only the activated form of Bax: red) or (**B**) monoclonal anti-cyt c (red). Laser confocal analyses were done as described in the material and methods section. (**C, D**) Human MSCs were plated into 6-well plates and 24 h later cultured in the absence or presence of 50 µg/ml etoposide (Eto). 24 h later the cells were either cultured in the presence of complete media (**C**) or in osteogenic differentiation media (**D**). At the different times indicated the cells were trypsinized and the number of viable cells determined by trypan blue exclusion.

The change in the conformation of Bax, mitochondrial integration and the subsequent release of cytochrome c (cyt c) are landmarks of apoptosis [12]. As shown in **Figure 3A**, an etoposide-treatment, even at a low concentration (i.e. 50 µg/ml) triggered an apoptotic change in conformation in Bax in hMSCs as illustrated by the binding to Bax by the anti-conformational monoclonal antibody 6A7 [12]. The release of cyt c from mitochondria is a major step in the execution of apoptosis that occurs downstream of Bax activation and mitochondrial association [12]. Using laser confocal microscopy as previously described [23], we analyzed the subcellular localization of cyt c in control and etoposide-treated hMSCs As illustrated in **Figure 3B**, no change in the localization of cyt c was observed in etoposide-treated cells, which remained mitochondrial as observed in control untreated cells. These results suggest that the blockade of apoptosis in undifferentiated hMSCs occurred at the mitochondrial level after Bax activation but before cyt c release. To analyze, the impact of etoposide treatment on hMSCs growth and differentiation, osteogenic differentiation was induced in control and etoposide-treated MSC. As shown in F**igure 3C**, in the absence of osteogenic differentiation, control and etoposide-treated MSC proliferated at the same rate for several days (i.e. up to 6 days *in vitro*). However, when differentiation was induced after etoposide treatment, hMSCs stopped proliferating and died after 2 days (**Figure 3D**). This result suggests that although undifferentiated hMSCs can sustain heavy DNA damage without the induction of apoptosis these cells cannot differentiate into mature cells.

### The capacity of hMSCs to undergo apoptosis is associated with differentiation

**Figure 4:**
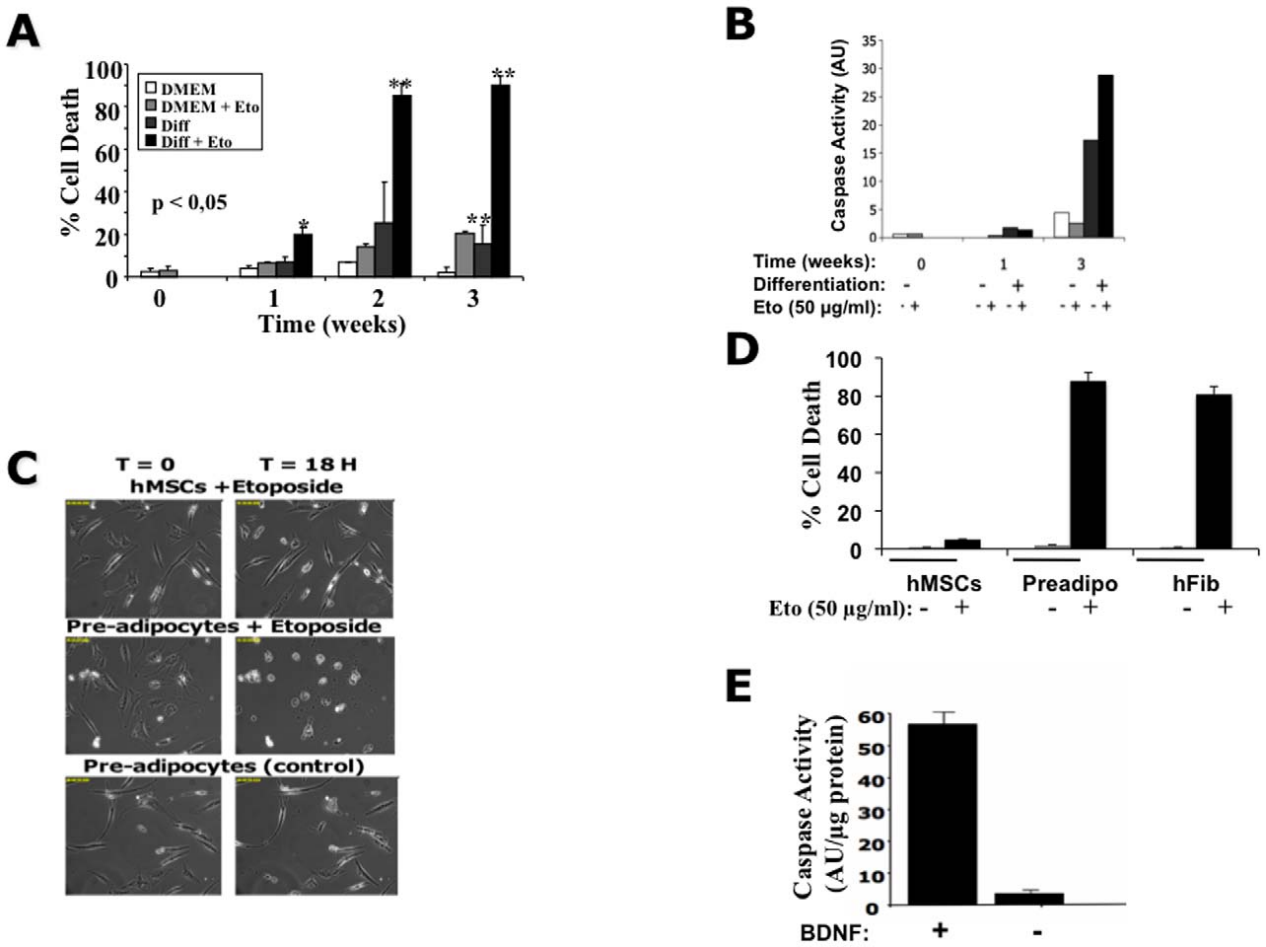
Sensitivity to cell death was acquired after induction of differentiation. (**A**) Human MSCs were cultured in complete or osteogenic differentiation medium for 0 to 3 weeks; in the absence or in the presence of 50 µg/ml etoposide (Eto). The number of viable cells was quantified by Trypan blue exclusion counting a minimum of 200 cells per condition. The data presented represent three independent experiments. (**B**) The cells treated as in (**A**) were collected and 10 µg whole cell lysates were assayed for caspase activity using Ac-DEVD-AMC as a substrate. The results are expressed as arbitrary units of caspase activity per µg protein. (**C**) Induction of apoptosis in hMSCs and adipocytes. Pictomicrographs of adipogenic differentiated hMSCs and hMSCs cultured in the absence or in the presence of 50 µg/ml etoposide (Eto) for 18 h. Images are representative of 4 independent experiments. (**D**) The number of cell death in hMSCs, preadipocyte (preadipo) or human fibroblast (hfib) cultures treated or not with 50 µg/ml etoposide (Eto) for 24 h was determined by Trypan blue exclusion (Countess), counting about 200 cells under each condition. The data are representative of 3 independent experiments. (**E**) Human MSCs induced to differentiate along the neuronal pathway (+10 µg/ml BDNF) or not (without BDNF) were cultured in the presence of 50 µg/ml etoposide (Eto) for 24 h before being assayed for caspase activity using Ac-DEVD-AMC as a substrate. The results are expressed as arbitrary units of caspase activity per µg protein.

The latter results suggested that the differentiation process was accompanied by a change in the sensitivity toward apoptosis. To address this point, hMSCs were cultured in osteogenic differentiation media for 1 to 3 weeks then treated or not with etoposide. Cell death was quantified by Trypan blue exclusion 24 h after the treatment. We found that the initiation of differentiation sensitized these cells to cell death. Furthermore, as early as one week after the induction of differentiation, etoposide was able to induce an effective cell death (**Figure 4A**). Apoptosis was identified by the measure of caspase activity (**Figure 4B**) and analysis with APO 2.7, which both are specific of apoptotic cells [26] (**Figure S1**).

We next investigated the sensitivity of hMSCs toward cell death upon the induction of differentiation along the adipogenic or neuronal pathways. Cells were differentiated then subjected to an etoposide treatment. **Figure 4C** illustrates cells engaged in adipogenic differentiation treated or not with etoposide. In agreement with our previous observations, only cells engaged along the adipogenic differentiation pathway were as sensitive to cell death as human fibroblasts (**Figure 4D**). Similarly, hMSCs cultured in the presence of bFGF/EGF and then BDNF to induce neural differentiation were responsive to etoposide at a concentration, which did not affect the cells treated only with bFGF/EGF (**Figure 4E**). Note that the treatment with etoposide was done in the absence of BDNF since BDNF is a survival factor and prevents cell death when present in the medium (**data not shown**). The decrease in cell viability was due to an increase in apoptosis as observed by the induction of caspase activity under these conditions (**Figure 4E**). Consistent with these results, we found that Bax was translocated to the mitochondria and cyt c was released from the mitochondria in etoposide-treated differentiated cells (**Figure S2**).

### Differential expression of Bcl-2 and Bcl-Xl between undifferentiated and differentiated hMSCs

**Figure 5:**
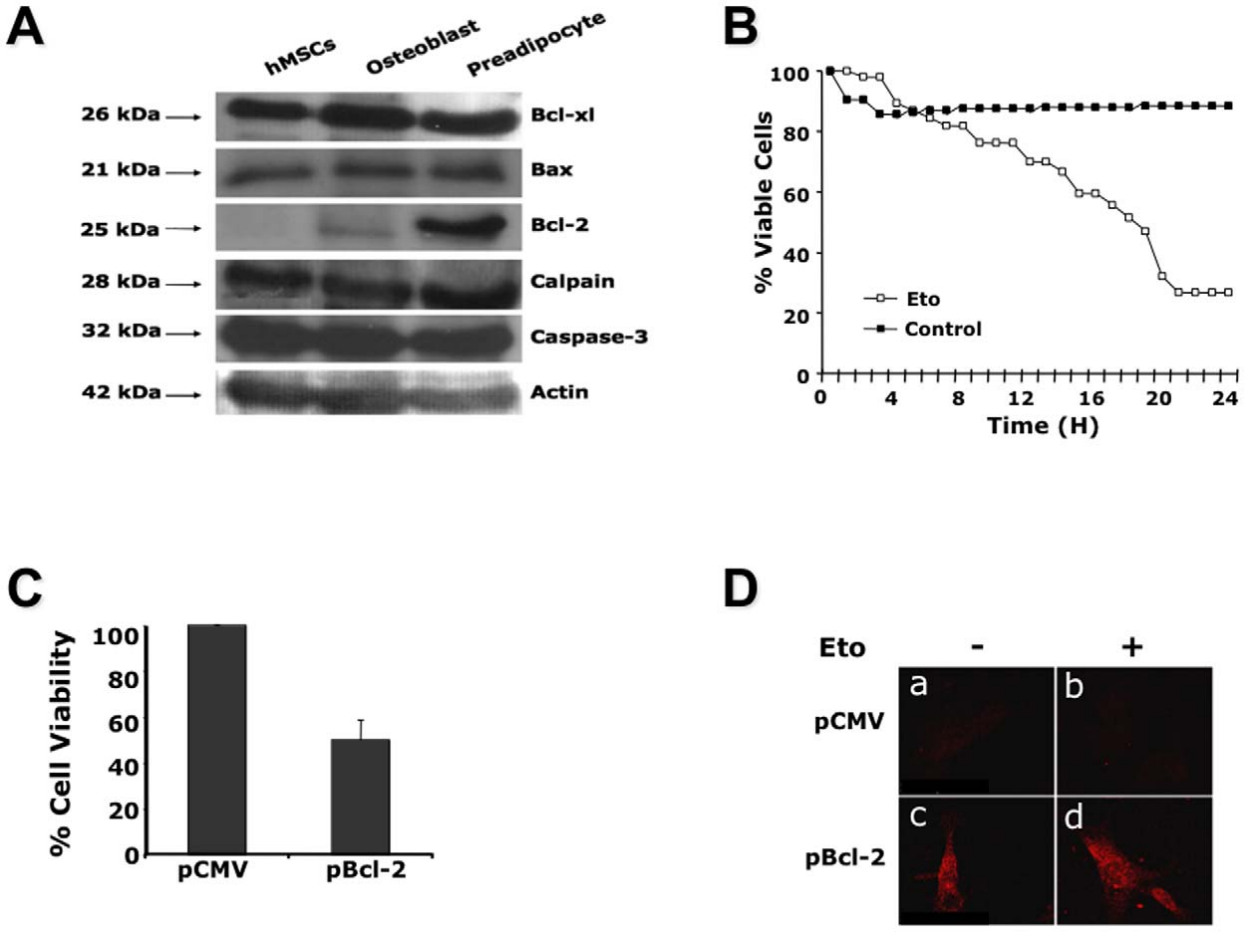
Expression of key components of the apoptotic machinery in hMSCs. (**A**) Western blot analyses of some of the main components of the apoptotic machinery. Total protein extracts were performed and 50 µg protein was analyzed on a 12% SDS-PAGE. Immunodetections were performed with the antibodies cited in the material and methods section. (**B**) The sensitivity of the hMSCs-shBcl-Xl-501 and hMSCs-shscr to apoptosis was determined by culturing the cells in the absence or in the presence of 50 µg/ml etoposide (Eto). The cells were analyzed over 48 h using video-microscopy with an acquisition every 10 min. The number of dead cells was determined at each time point and rounded up for every hour. The results are presented as the percentage of dead cells in treated cultures versus untreated cultures. The number of cells analyzed was about 100 per condition. The results are representative of three independent experiments. (**C**) Cells were nucleofected with either pCMV or pBcl-2 and 24 h later cell viability was determined by Trypan blue exclusion. (**D**) Twenty-four hours after the transfection of hMSCs with pCMV (a, b) or pBcl-2 (c, d); these cells were cultured in the absence (a, c) or presence (b, d) of 50 µg/ml etoposide (Eto) then analyzed for the expression of active caspase 3 (red) by confocal microscopy. The data presented are representative of 2 independent experiments.

The fact that caspase activity could not be detected in undifferentiated hMSCs upon etoposide, UV or STS treatments whereas Bax was “activated” in both undifferentiated and differentiated cells suggests that the mitochondrial apoptotic pathway was impaired in undifferentiated cells at the mitochondrial level. Thus, we analyzed the expression of key proteins of the apoptotic program in these cells. As shown in **Figure 5A**, immunoblots revealed that hMSCs expressed key components of apoptosis belonging to the BCL-2 family such as the anti-apoptotic protein Bcl-Xl and the pro-apoptotic protein Bax. Mcl-1 and Bak were also found in both undifferentiated and differentiated cells (**data not shown**). However, undifferentiated hMSCs lack the expression of Bcl-2. On the other hand, hMSCs also expressed proteins essential for the execution phase of apoptosis such as caspase 3 (**Figure 5A**) and caspase 7, 8 and 9 **(data not shown**). Quite remarkably, Bcl-2 was expressed in differentiated cells although to a lesser degree in osteoblasts than in adipocytes (**Figure 5A**). Indeed, it has been reported that primitive human hematopoietic precursors (i.e. CD34^+^/lin^−^) express Bcl-Xl but not Bcl-2 [27] and that this differential expression influence their lineage choice [13].

We addressed the question of the involvement of Bcl-Xl and Bcl-2 in the survival and/or differentiation of hMSCs. First we down-regulated the expression of Bcl-Xl by shRNA (**Figure S3** illustrates the efficiency of the shRNA Bcl-Xl). The knock-down of Bcl-Xl in hMSCs made these cells susceptible to low concentrations of etoposide (i.e. 50 µg/ml) that was not able to trigger apoptosis in hMSCs infected with a scr-shRNA (**Figure 5B**). This result suggests that Bcl-Xl plays an essential role in protecting hMSCs against apoptosis. On the other hand, the over-expression of Bcl-2 (**Figure S4**) induced a massive cell death in undifferentiated hMSCs and this without the addition of apoptotic inducers (**Figure 5C**). To underline this observation, hMSCs transfected with Bcl-2, treated or not with etoposide, exhibited caspase 3 activity while no activity was detected in mock-transfected cells treated or not with etoposide (**Figure 5D**).

### Bcl-2 induces apoptosis in undifferentiated hMSCs though its interaction with Nur 77

**Figure 6:**
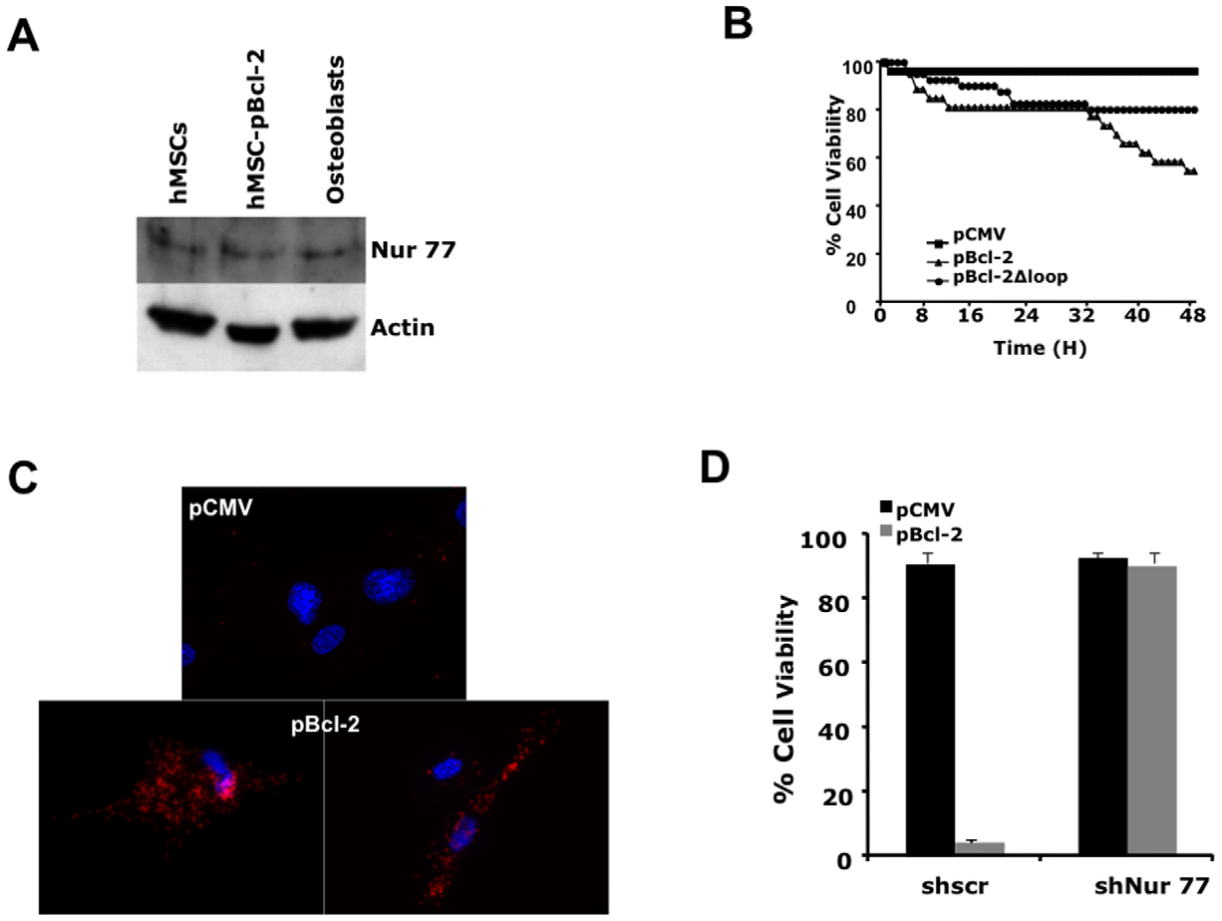
Interaction between Bcl-2 and Nur 77. (**A**) Western blot analyses of Nur 77 expression in hMSCs, hMSCs transfected with pBcl-2 and osteoblasts. This is a representative immunoblot of 2 different experiments. (**B**) Cells were transfected with plasmids: pCMV, pCMV containing Bcl-2 (pBcl-2) or Bcl-2 deleted in the amino acids 30–85 (pBcl-2Δloop). 16 h post-transfection the cells were plated in 6-well plated and video-filmed over 48 h with acquisition every 10 min. The number of dead cells was determined at each time point and rounded up for every hour. The results are presented as the percentage of dead cells in treated cultures versus untreated cultures. The number of cells analyzed was minimum 100 per condition. The results are representative of 3 independent experiments. (**C**) A PLA between Bcl-2 and Nur 77 was performed on hMSCs transfected with pCMV or pBcl-2 as described in the materials and methods section. Fluorescence was detected using the fluorophore Tye-563 that excites at 557 nm and emits at 563 nm. The nuclei were stained with Dapi present in the mounting solution (Prolong® Gold anti-fade). Spots indicated interaction between Nur77 and Bcl-2. These experiments were repeated 3 times, each performed in duplicate. (**D**) Human MSCs infected with either shNur77 or shscr were then transfected with pCMV or pBcl-2 and then treated with 50 µg/ml etoposide. Note that for all experiments only Nur77-25 was used. Cell viability was determined by Trypan blue exclusion, 24 h after treatments. The results are representative of 3 different experiments done in triplicate.

Recently, Bcl-2 has been shown to be transformed into a pro-apoptotic protein upon its binding to Nur 77 (also known as TR3 or NGFI-B) through the Bcl-2 loop region [28]. Nur 77 is present in hMSCs and there is no increase in its expression during osteogenic differentiation (**Figure 6A**). The expression of Bcl-2 in undifferentiated hMSCs but not that of a mutant lacking the binding domain with Nur 77 (i.e. Bcl-2 loop region) induced apoptosis in these cells (**Figure 6B**). Data from the proximity ligation assay (PLA) on hMSCs transfected with either pCMV or pBcl-2 suggested that Bcl-2 and Nur 77 were in very close proximity to each other in cells transfected by pBcl-2 as visualized by the number of fluorescent dots present in these cell (**Figure 6C**). Nur 77 is present in both the cytoplasmic and nuclear compartments in undifferentiated hMSCs but is mostly mitochondrial in hMSCs (**Figure S5A**). In Bcl-2 transfected hMSCs, there was a 50% increase in the amount of Nur 77 inserted into the mitochondria as compared to pCMV-transfected cells (**Figure S5B**). A shRNA down-regulation of Nur 77 was performed in undifferentiated hMSCs (**Figure S6**). The knock-down of Nur 77 by ShRNA (ShNur 77-25) decreased significantly the Bcl-2-induced cell death in undifferentiated hMSCs as compared to cells transfected with pCMV (**Figure 6D**). Note that a scrambled ShRNA did not affect Bcl-2 induced cell death (**Figure 6D**). Similar results were obtained with a ShNur 77 (i.e. ShNur 77-26) that caused no alteration in Nur 77 expression (**data not shown**). Taken together, our results suggest that apoptosis induced by ectopic Bcl-2 in undifferentiated hMSCs is induced by its interaction with Nur 77.

## Discussion

In this study, we have analyzed the relationship between sensitivity to cell death and differentiation of hMSCs. We have found that undifferentiated hMSC were highly resistant to apoptosis until they engaged along a differentiation pathway. The hMSCs used in this study were isolated from middle-aged donors; were short-term cultures and used for a limited number of passages to avoid the interference with senescence (**Figure 1**). In this study, we show that hMSCs are resistant to potent apoptosis inducers at concentrations that otherwise kill cancer cells such as the K562 cells or human GBM cells as well as primary cultures of human fibroblasts (**Figure 2**). The nature of this intrinsic resistance to apoptosis implies the inhibition of the mitochondrial intrinsic pathway as demonstrated by the lack of cyt c release upon the induction of apoptosis in hMSCs (**Figure 3**). Human MSCs that have undergone DNA damage during etoposide treatment, survive and proliferate but die upon differentiation (**Figure 3**). However, it should be noted that this treatment might not reflect the type of DNA damage that could occur under normal physiological conditions. However, one could postulate that these features are involved in the elimination of damaged hMSCs. Of note, upon addition of differentiating factors, hMSCs shift their sensitivity toward apoptosis inducers (**Figure 4**). This feature is not restricted to osteogenic lineage as it can be also observed during neural and adipocytic differentiation as well (**Figure 4**).

One important finding in this work is the differential expression of Bcl-2 between differentiated and undifferentiated hMSCs (**Figure 5**). The control of Bcl-2 versus cell differentiation has been shown in several cell types, including hematopoietic, neural and epithelial cells [13]. Low levels of Bcl-2 are found in immature cells and high levels in mature cells in the lymphoid compartment [29]. Similarly, Bcl-2 expression is up-regulated during the differentiation of hematopoietic progenitors. Our results strengthen the hypothesis of a link between low Bcl-2 levels and “stemness” outside the hematopoietic compartment. In hMSCs this effect appears to be different from its anti-apoptotic role, which is apparently carried out by Bcl-Xl (**Figure 5**). The latter feature is consistent with previous studies that show that Bcl-Xl functions as a prime regulator of the viability of immature cells during the development of the nervous and hematopoietic system [30]. However, in this study we have analyzed the behavior of isolated hMSCs, a condition that does not necessarily mirror a physiological situation.

On the other hand, Bcl-2 induces cell death when expressed in undifferentiated hMSCs though its interaction with Nur 77 (**Figure 6**). Nur 77 is an orphan member of the nuclear receptor subfamily 4 that is expressed in many cell types where it plays several key roles including induction of apoptosis and control of differentiation. Inhibition of differentiation by Nur 77 through its transcriptional targets has been documented in adipocytes [31]. On the other hand, it has been shown to induce cell cycle arrest and differentiation in dopaminergic cells [32]. During TCR-mediated clonal deletion, Nur 77 appears to be instrumental through its capacity to induce apoptosis. It is thought that the translocation of Nur 77 to the cytoplasm promotes cell death, while its retention in the nucleus promotes survival and proliferation through transcriptional activity but also via protein-protein interactions. Two of Nur 77 primary interactive targets are Bcl-2 and Bcl-B, the binding to which induces a drastic change, switching their function from anti-apoptotic to pro-apoptotic [28]. However, Nur 77 is not capable of interacting with either Bcl-Xl or Mcl-1 [33]. The interaction between Nur 77 and Bcl-2 is instrumental in the induction of apoptosis during negative selection of autoreactive thymocytes [34]. Here, we show a parallel mechanism in hMSCs, demonstrating that Bcl-2 is down-regulated in undifferentiated hMSCs and this down-regulation preserves these cells from cell death via apoptosis. These results suggest that a complex cross-regulation between Bcl-2 and Nur 77 controls the survival and the differentiation of hMSCs through a mechanism similar to that observed during the negative selection in the immune system [35].

A better understanding of the different mechanisms that regulate apoptosis in hMSCs could provide important clues on the relationship between the cell death program and the differentiation programs in adult stem cells. This could have important consequences on both cancer and regenerative medicine.

## Supporting Information

Figure S1 **Human MSCs were cultured in complete or osteogenic differentiation medium for 0, 1 or 3 weeks and in the absence or presence of 50 µg/ml etoposide (Eto).** The cells were trypsinized and the number of apoptotic cells was labelled with APO 2.7-PE and then quantified by cytometry. The results are representative of three independent experiments.
(TIF)

Figure S2 **Western blot analyses of Bax and cyt c in cytoplasmic and mitochondrial fractions from hMSCs treated or not with 50 µg/ml etoposide.**
(TIF)

Figure S3 **Western blot analyses of hMSCs infected with sh-scr and shBcl-Xl-501 showing the Knock-down of Bcl-Xl.**
(TIF)

Figure S4 **Western blot analyses of hMSCs transfected with pCMV and pBcl-2 showing the expression of Bcl-2.**
(TIF)

Figure S5 (**A**) The localization of Nur 77 in hMSCs nucleofected with pBcl-2 or pCMV was determined by confocal microscopy. These are representative pictures of 3 different experiments. (**B**) Calculation of the percentage of co-localization of Nur 77 (green) with F1-ATPase (mitochondria: red) in hMSCs transfected with either pCMV or pBcl-2 using Metamorph 7.5.6. The algorithm «XOR» was applied to Nur 77 and the «AND» algorithm was used to threshold the non-colocalized Nur 77. The results are the mean of 10 views from 3 different experiments.
(TIF)

Figure S6 **Western blot analyses of hMSCs infected with sh-scr or shNur 77 showing the different levels of knock-down of Nur 77 using the different shNur 77 viral particles.** Note for the experiments Nur 77-25 was used.
(TIF)

Table S1 **List of antibodies used to phenotype the hMSCs by FACS analyses.**
(TIF)

## References

[1] He S, Nakada D, Morrison SJ (2009). Mechanisms of stem cell self-renewal.. Ann Rev Cell Dev Biol.

[2] Reya T, Morrison SJ, Clarke MF, Weissman IL (2001). Stem cells, cancer, and cancer stem cells.. Nature.

[3] Caplan AI (2007). Why are MSCs therapeutic? New data: new insight.. J Pathol.

[4] Uccelli A, Moretta L, Pistoia V (2008). Mesenchymal stem cells in health and disease.. Nat Rev Immunol.

[5] Phinney DG, Prockop DJ (2007). Concise review: mesenchymal stem/multipotent stromal cells: the
state of transdifferentiation and modes of tissue repair–current
views.. Stem Cells.

[6] Valtieri M, Sorrentino A (2008). The mesenchymal stromal cell contribution to
homeostasis.. J Cell Physiol.

[7] Streetz KL, Doyonnas R, Grimm D, Jenkins DD, Fuess S (2008). Hepatic parenchymal replacement in mice by transplanted
allogeneic hepatocytes is facilitated by bone marrow transplantation and
mediated by CD4 cells.. Hepatology.

[8] Kennea NL, Stratou C, Naparus A, Fisk NM, Mehmet H (2005). Functional intrinsic and extrinsic apoptotic pathways in human
fetal mesenchymal stem cells.. Cell Death Diff.

[9] Li W, Ma N, Ong LL, Nesselmann C, Klopsch C (2007). Bcl-2 engineered MSCs inhibited apoptosis and improved heart
function.. Stem Cells.

[10] Li J, Law HK, Lau YL, Chan GC (2004). Differential damage and recovery of human mesenchymal stem cells
after exposure to chemotherapeutic agents.. Br J Haematol.

[11] Mueller LP, Luetzkendorf J, Mueller T, Reichelt K, Simon H (2006). Presence of mesenchymal stem cells in human bone marrow after
exposure to chemotherapy: evidence of resistance to apoptosis
induction.. Stem Cells.

[12] Youle RJ, Strasser A (2008). The BCL-2 protein family: opposing activities that mediate cell
death.. Nat Rev Mol Cell Biol.

[13] Haughn L, Hawley RG, Morrison DK, von Boehmer H, Hockenbery DM (2003). BCL-2 and BCL-XL restrict lineage choice during hematopoietic
differentiation.. J Biol Chem.

[14] Pittenger MF, Mackay AM, Beck SC, Jaiswal RK, Douglas R (1999). Multilineage potential of adult human mesenchymal stem
cells.. Science.

[15] Ishii M, Koike C, Igarashi A, Yamanaka K, Pan H (2005). Molecular markers distinguish bone marrow mesenchymal stem cells
from fibroblasts.. Biochem Biophys Res Commun.

[16] Pontikoglou C, Delorme B, Charbord P (2008). Human bone marrow native mesenchymal stem cells.. Regen Med.

[17] Bonab MM, Alimoghaddam K, Talebian F, Ghaffari SH, Ghavamzadeh A (2006). Aging of mesenchymal stem cell in vitro.. BMC Cell Biol.

[18] Sharpless NE, DePinho RA (2007). How stem cells age and why this makes us grow
old.. Nat Rev Mol Cell Biol.

[19] Stenderup K, Justesen J, Clausen C, Kassem M (2003). Aging is associated with decreased maximal life span and
accelerated senescence of bone marrow stromal cells.. Bone.

[20] Wagner W, Horn P, Castoldi M, Diehlmann A, Bork S (2008). Aging and replicative senescence have related effects on human
stem and progenitor cells.. PLoS ONE.

[21] Oguro H, Iwama A (2007). Life and death in hematopoietic stem cells.. Curr Opin Immunol.

[22] Batista LF, Kaina B, Meneghini R, Menck CF (2009). How DNA lesions are turned into powerful killing structures:
insights from UV-induced apoptosis.. Mutat Res.

[23] Cartron PF, Juin P, Oliver L, Martin S, Meflah K (2003). Nonredundant role of Bax and Bak in Bid-mediated
apoptosis.. Mol Cell Biol.

[24] Zhu W, Chen J, Cong X, Hu S, Chen X (2006). Hypoxia and serum deprivation-induced apoptosis in mesenchymal
stem cells.. Stem Cells.

[25] Ren H, Cao Y, Zhao Q, Liao L, Jia M (2006). Proliferation and differentiation of bone marrow stromal cells
under hypoxic conditions.. Biochem Biophys Res Com.

[26] Koester SK, Roth P, Mikulka WR, Schlossman SF, Zhang C (1997). Monitoring early cellular responses in apoptosis is aided by the
mitochondrial membrane protein-specific monoclonal antibody
APO2.7.. Cytometry.

[27] Perk JR, Bernstein ID, Hockenbery DM (1995). Primitive human hematopoietic precursors express Bcl-x but not
Bcl-2.. Blood.

[28] Lin B, Kolluri SK, Lin F, Liu W, Han YH (2004). Conversion of Bcl-2 from protector to killer by interaction with
nuclear orphan receptor Nur77/TR3.. Cell.

[29] Orelio C, Dzierzkak E (2007). Bcl-2 expression and apoptosis in the regulation of hematopoietic
stem cells.. Leuk Lymph.

[30] Motoyama N, Wang F, Roth KA, Sawa H, Nakayama K (1995). Massive cell death of immature hematopoietic cells and neurons in
Bcl-x-deficient mice.. Science.

[31] Chao LC, Bensinger SJ, Villanueva CJ, Wroblewski K, Tontonoz P (2008). Inhibition of adipocyte differentiation by Nur77, Nurr1, and
Nor1.. Mol Endocrinol.

[32] Lévesque D, Rouillard C (2007). Nur77 and retinoid X receptors: crucial factors in
dopamine-related neuroadaptation.. Trends Neurosci.

[33] Luciano F, Krajewska M, Ortiz-Rubio P, Krajewski S, Zhai D (2007). Nur77 converts phenotype of Bcl-B, an antiapoptotic protein
expressed in plasma cells and myeloma.. Blood.

[34] Sohn SJ, Thompson J, Winoto A (2007). Apoptosis during negative selection of autoreactive
thymocytes.. Curr Opin Immunol.

[35] Thompson J, Winoto A (2008). During negative selection, Nur77 family proteins translocate to
mitochondria where they associate with Bcl-2 and expose its proapoptotic BH3
domain.. J Exp Med.

