# Supplementary figures and images for: Distinct Roles of Bcl-2 and Bcl-Xl in the Apoptosis of Human Bone Marrow Mesenchymal Stem Cells during Differentiation
Source: PLoS One. 2011 May 12;6(5):e19820. doi: 10.1371/journal.pone.0019820 (PMC3093403; doi:10.1371/journal.pone.0019820)

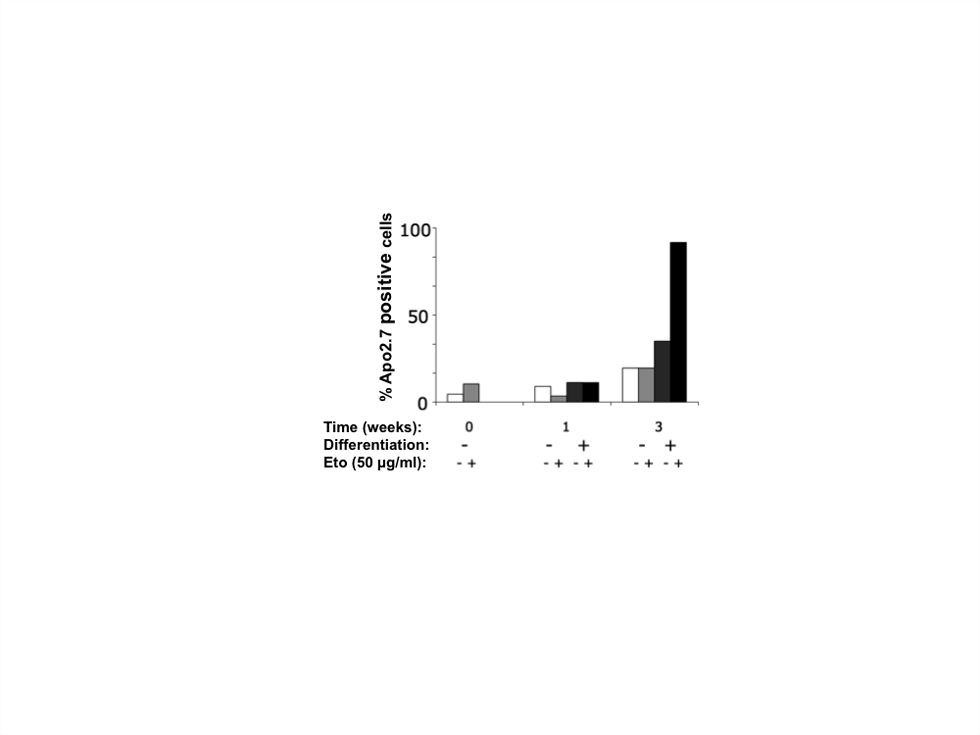

Supplement: Figure S1 — Human MSCs were cultured in complete or osteogenic differentiation medium for 0, 1 or 3 weeks and in the absence or presence of 50 µg/ml etoposide (Eto). The cells were trypsinized and the number of apoptotic cells was labelled with APO 2.7-PE and then quantified by cytometry. The results are representative of three independent experiments. (TIF) [file pone.0019820.s001.tif]

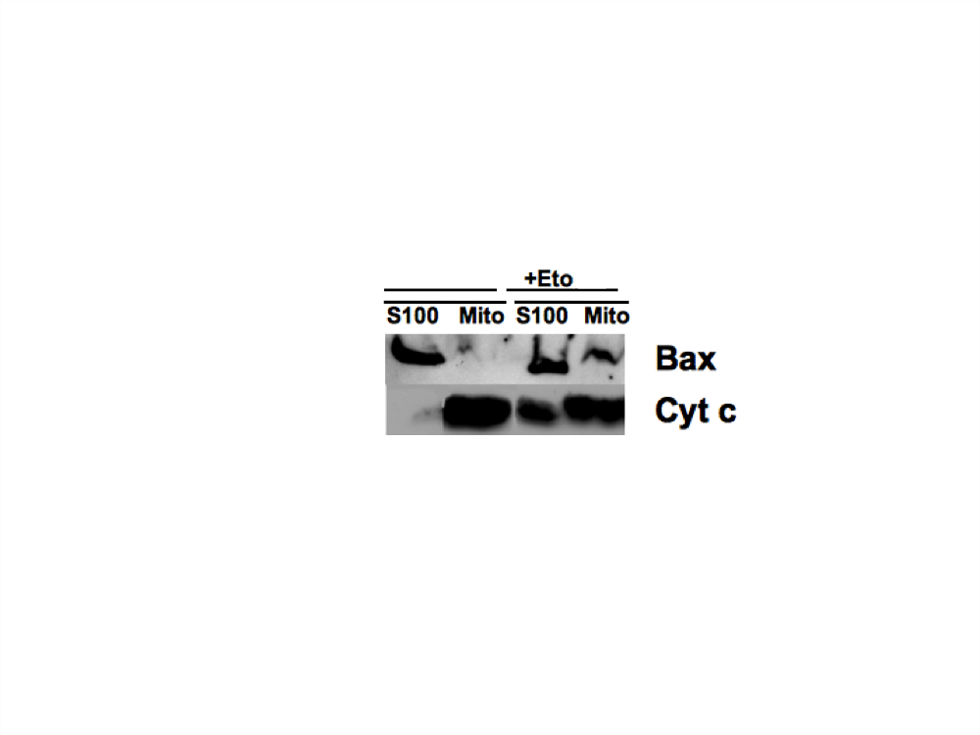

Supplement: Figure S2 — Western blot analyses of Bax and cyt c in cytoplasmic and mitochondrial fractions from hMSCs treated or not with 50 µg/ml etoposide. (TIF) [file pone.0019820.s002.tif]

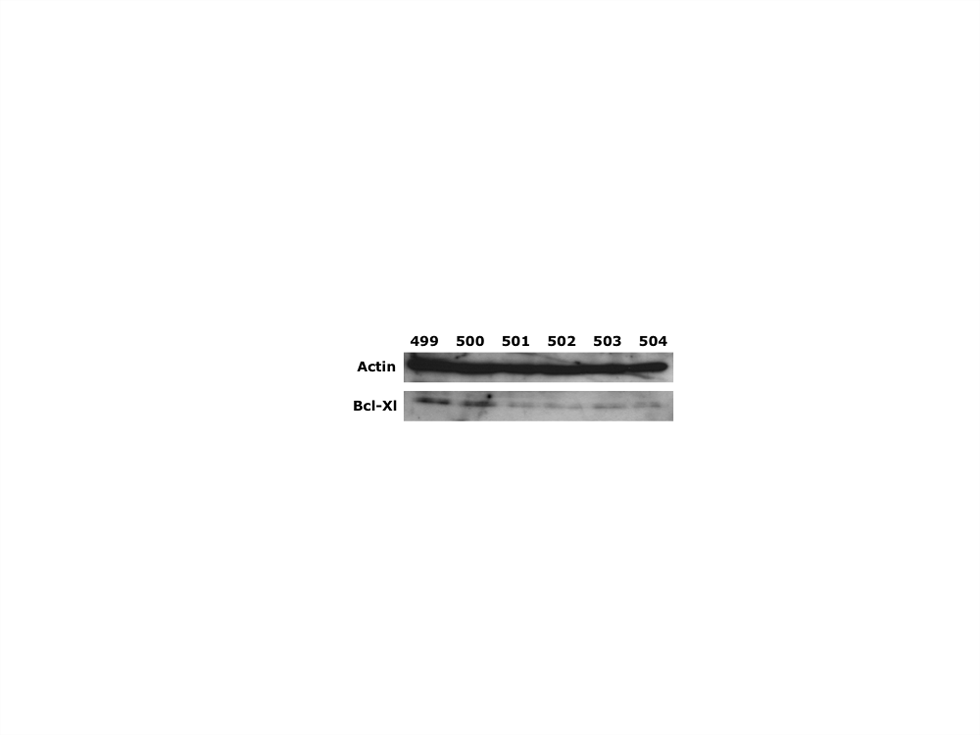

Supplement: Figure S3 — Western blot analyses of hMSCs infected with sh-scr and shBcl-Xl-501 showing the Knock-down of Bcl-Xl. (TIF) [file pone.0019820.s003.tif]

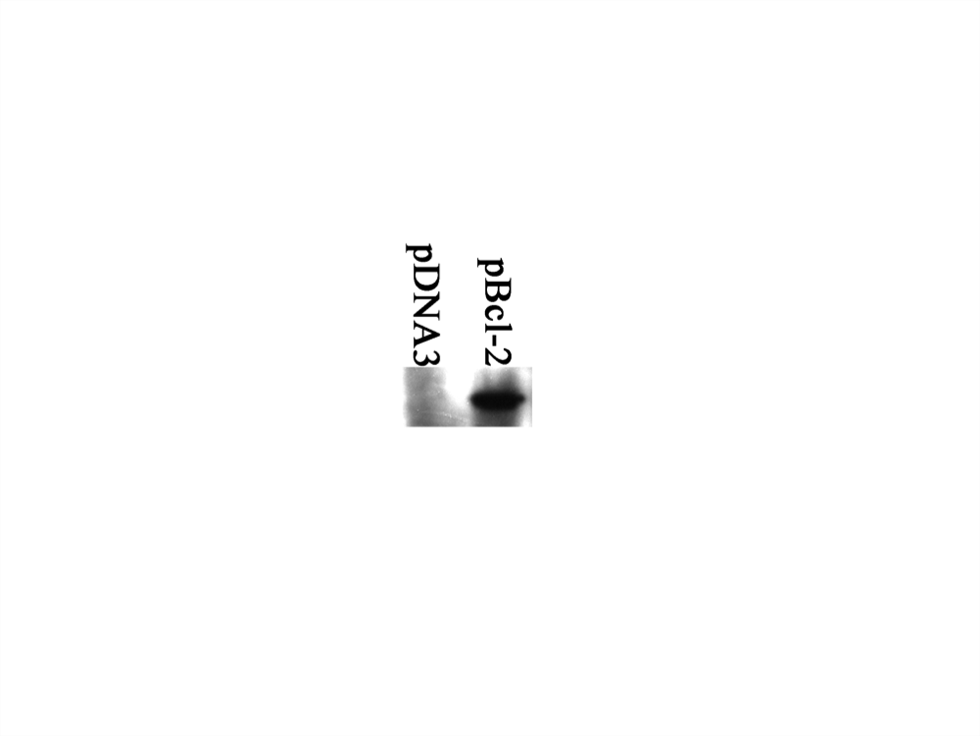

Supplement: Figure S4 — Western blot analyses of hMSCs transfected with pCMV and pBcl-2 showing the expression of Bcl-2. (TIF) [file pone.0019820.s004.tif]

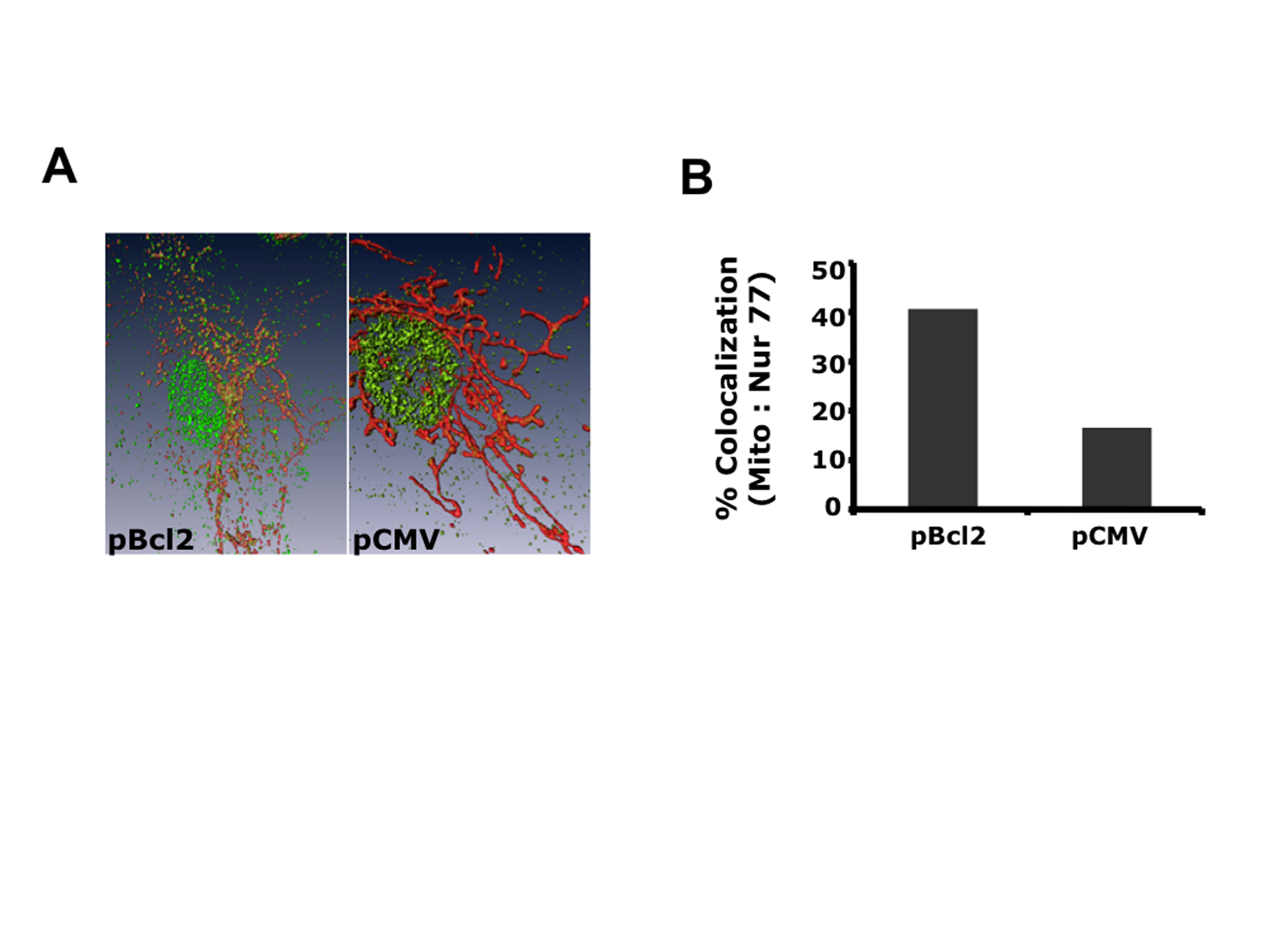

Supplement: Figure S5 — (A) The localization of Nur 77 in hMSCs nucleofected with pBcl-2 or pCMV was determined by confocal microscopy. These are representative pictures of 3 different experiments. (B) Calculation of the percentage of co-localization of Nur 77 (green) with F1-ATPase (mitochondria: red) in hMSCs transfected with either pCMV or pBcl-2 using Metamorph 7.5.6. The algorithm «XOR» was applied to Nur 77 and the «AND» algorithm was used to threshold the non-colocalized Nur 77. The results are the mean of 10 views from 3 different experiments. (TIF) [file pone.0019820.s005.tif]

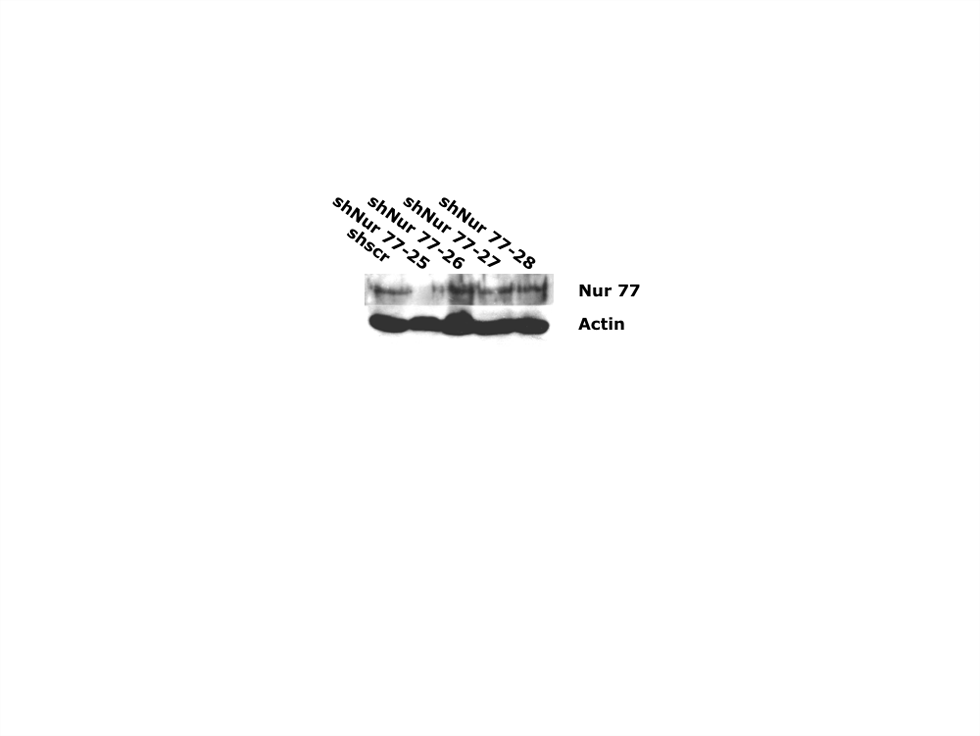

Supplement: Figure S6 — Western blot analyses of hMSCs infected with sh-scr or shNur 77 showing the different levels of knock-down of Nur 77 using the different shNur 77 viral particles. Note for the experiments Nur 77-25 was used. (TIF) [file pone.0019820.s006.tif]

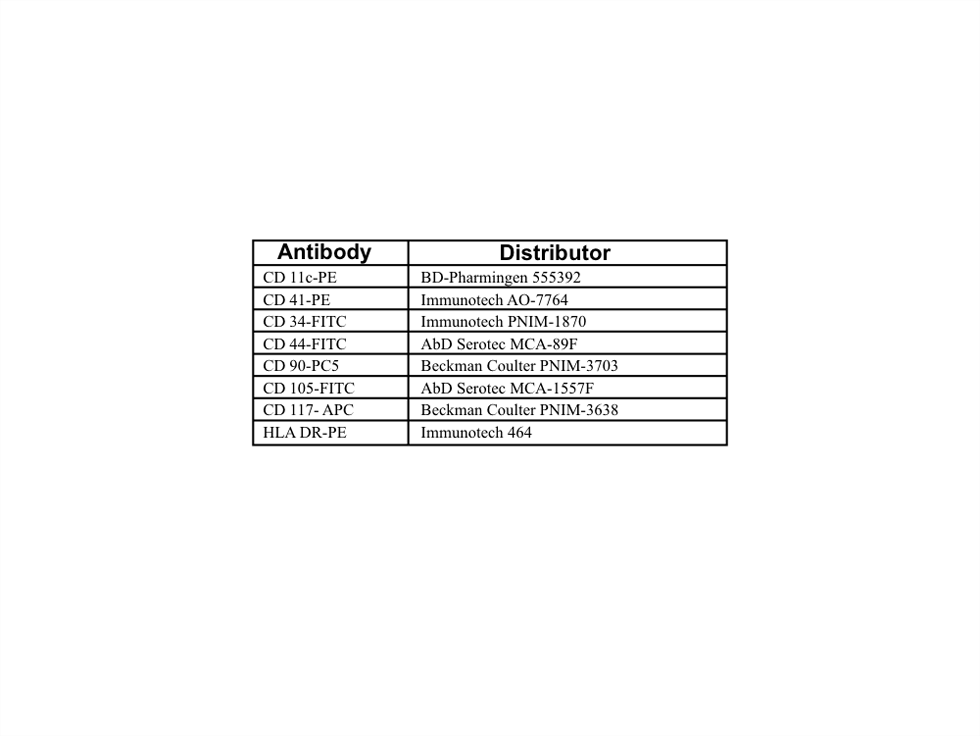

Supplement: Table S1 — List of antibodies used to phenotype the hMSCs by FACS analyses. (TIF) [file pone.0019820.s007.tif]
